# Chromium Recovery from Chromium-Loaded *Cupressus lusitanica* Bark in Two-Stage Desorption Processes

**DOI:** 10.3390/plants12183222

**Published:** 2023-09-10

**Authors:** Alma Rosa Netzahuatl-Muñoz, Erick Aranda-García, Eliseo Cristiani-Urbina

**Affiliations:** 1 Departamento de Ingeniería Bioquímica, Instituto Politécnico Nacional, Escuela Nacional de Ciencias Biológicas, Avenida Wilfrido Massieu s/n, Unidad Profesional Adolfo López Mateos, Delegación Gustavo A. Madero, Mexico City 07738, Mexico; 2 Programa de Ingeniería en Biotecnología, Universidad Politécnica de Tlaxcala, Avenida Universidad Politécnica No. 1, Colonia San Pedro Xalcaltzinco, Tepeyanco, Tlaxcala 90180, Mexico

**Keywords:** desorption, kinetics, hexavalent chromium, total chromium, *Cupressus lusitanica* bark, eluent

## Abstract

Hexavalent chromium (Cr(VI)) contamination poses serious health and environmental risks. Chromium biosorption has been employed as an effective means of eradicating Cr(VI) contamination. However, research on chromium desorption from chromium-loaded biosorbents is scarce despite its importance in facilitating industrial-scale chromium biosorption. In this study, single- and two-stage chromium desorption from chromium-loaded *Cupressus lusitanica* bark (CLB) was conducted. Thirty eluent solutions were evaluated first; the highest single-stage chromium desorption efficiencies were achieved when eluent solutions of 0.5 M NaOH, 0.5 M H_2_SO_4_, and 0.5 M H_2_C_2_O_4_ were used. Subsequently, two-stage kinetic studies of chromium desorption were performed. The results revealed that using 0.5 M NaOH solution in the first stage and 0.5 M H_2_C_2_O_4_ in the second stage enabled the recovery of almost all the chromium initially bound to CLB (desorption efficiency = 95.9–96.1%) within long (168 h) and short (3 h) desorption periods at each stage. This study clearly demonstrated that the oxidation state of the recovered chromium depends on the chemical nature and concentration of the eluent solution. The results suggest the possible regeneration of chromium-loaded CLB for its subsequent use in other biosorption/desorption cycles.

## 1. Introduction

Hexavalent chromium (Cr(VI)) contamination is among the most critical anthropogenic environmental problems at present [1,2,3]. Cr(VI) is present in liquid effluents generated by several manufacturing and industrial processes, including leather tanning, dyestuffs, textile dyeing, mining, and electroplating [4,5,6]. Cr(VI) causes serious public health and environmental problems due to its extremely high toxicity, mutagenicity, carcinogenicity, and teratogenicity [1,4,5,7,8].

Although various physical, chemical, biological, and combined remediation technologies exist to detoxify Cr(VI)-contaminated wastewater, biosorption has recently garnered much attention due to its great potential to effectively detoxify Cr(VI)-contaminated wastewaters and substantially reduce the harmful risks of Cr(VI) on the natural and social environment [5,6,9,10,11,12]. Chromium biosorption from Cr(VI) solutions has been extensively investigated. The biological materials that have been used as biosorbents are diverse; the biomass of agricultural, forestry, and fishery wastes and byproducts, as well as the biomass from fermentation industries, exhibit the greatest potential as effective biosorbents because they are commonly available, inexpensive, and have high biosorption capacity [5,10,12,13,14,15].

The successful application of heavy-metal biosorption depends not only on the capacity and rate of a biosorbent to adsorb the heavy metal of interest from aqueous solutions but also on the ease of heavy-metal desorption and regeneration of the biosorbent [16,17]. The desorption of heavy metals from a metal-loaded biosorbent that has been used to treat metal-contaminated wastewater can be accomplished using suitable eluents; such an approach is important for metal recovery and regeneration, reuse, and safe disposal of the biosorbent, thereby reducing the amount of waste generated and the operating and capital costs of the wastewater treatment process [18,19]. The process conditions that enable the effective and efficient desorption of heavy metals from a loaded biosorbent must be established on a case-to-case basis because they depend mainly on the nature of the biosorbent and the type of interaction between the heavy metal and the biosorbent [20].

However, chromium desorption has not been comprehensively studied despite its importance for the successful implementation of chromium biosorption on an industrial scale. The available studies on chromium desorption have reported very low efficiencies; hence, it is necessary to use more concentrated eluting solutions [21,22] and/or increase the contact time between the chromium-loaded biosorbent and the eluting solution [23,24,25]. Moreover, some studies reported that the duration of chromium desorption was longer than that of chromium biosorption [26], which is unusual in biosorption/desorption processes.

The following facts must be taken into account in chromium desorption studies: (1) Chromium can be biosorbed on a biosorbent surface mainly as trivalent chromium (Cr(III)) and/or as hexavalent chromium (Cr(VI)), which are the most stable and abundant oxidation states of chromium in the natural environment [26,27,28,29,30,31]; part of the chromium biosorbed on coir pith was also found as pentavalent chromium (Cr(V)) [32]. (2) There are different interactions between biosorbed chromium species (for example, Cr(VI) and Cr(III)) and a biosorbent, including electrostatic attraction, ion exchange, and chemical complexation [33].

*Cupressus lusitanica* bark (CLB) is a readily available forestry waste that exhibits a remarkable ability to remove Cr(VI) and total chromium from Cr(VI) acid solutions. Moreover, based on the results of equilibrium, kinetic, and thermodynamic studies of chromium biosorption, as well as of diffuse-reflectance infrared Fourier-transform spectroscopy analysis, it is known that CLB biosorbs chromium through a four-step reaction mechanism: (1) the reaction between oxygen-containing functional groups and Cr(VI) oxyanions to form Cr(VI) complexes (biosorption of Cr(VI) oxyanions), (2) the bioreduction of Cr(VI) to Cr(III), (3) the generation of carboxyl functional groups via the oxidation of oxygen-containing groups, and (4) the reaction between Cr(III) ions and carboxyl groups to form Cr(III)–carboxylate complexes. Cr(III)–carboxylate complexes were shown to be the predominant form of the chromium biosorbed on CLB; however, it was not dismissed that Cr(III) formed during Cr(VI) bioreduction could form chemical complexes with some other functional groups (e.g., phenolic groups) present on the CLB surface [34,35].

Currently, CLB is among the most promising biosorbents for chromium biosorption from Cr(VI) solutions; consequently, there has been great technological and scientific interest to investigate its chromium desorption performance to improve the viability of chromium bioremediation processes. The objective of this study was to analyze the effect of various eluting solutions on the desorption of chromium from loaded CLB and their relationship with the oxidation state of the desorbed chromium. To attain this objective, eluent solution selection studies were initially conducted, followed by desorption kinetic studies in single and two stages. To the best of our knowledge, this is the first study that reports the efficient desorption of chromium in a two-stage process.

## 2. Results and Discussion

### 2.1. Selection of Eluent Solutions for Chromium Desorption from Chromium-Loaded CLB

The chromium-loaded CLB particles used in the desorption assays were found to contain 149 mg of total chromium per g of dry biosorbent.

Considering that the effect of eluent solutions on chromium desorption from chromium-loaded biosorbents depends mainly on the oxidation state of chromium biosorbed by the biosorbents (Cr(VI) and/or Cr(III)) and on the type of interaction between chromium and the biosorbents, various eluting solutions were evaluated in this study.

Table 1 shows the total chromium desorption efficiencies of the eluent solutions after 3 h of contact with chromium-loaded CLB. Metal desorption efficiencies were low—less than 14%. The eluent solutions in which the highest chromium desorption efficiencies were observed were acidic and alkaline solutions: 0.1 M KOH, 0.1 M NaOH, 0.1 M H_2_C_2_O_4_ (oxalic acid), and 0.1 M H_2_SO_4_. NaOH and KOH exhibited similar chromium desorption efficiencies at 0.01 and 0.1 M; this result is expected because both alkalis have similar properties and are almost interchangeable. NaOH, H_2_C_2_O_4_, and H_2_SO_4_ were, therefore, selected as eluent solutions for the subsequent experiments in this study.

Acidic and alkaline solutions are useful for the desorption of chromium biosorbed onto biosorbents through weak interactions, such as electrostatic attraction or ion exchange [36,37]. In this context, it has been reported that the protons of mineral acids such as HCl, H_2_SO_4_, and HNO_3_ can displace metals from the active sites of biosorbents [38]. In contrast, extensive screening of efficient desorbents is required when chromium is biosorbed onto biosorbents through chelation, complexation, and microprecipitation [37].

The low chromium desorption efficiencies obtained in this study are comparable to those obtained when low concentrations of acid or alkaline eluent solutions have been used for the desorption of chromium biosorbed on lignocellulosic materials, such as tamarind (*Tamarindus indica*) seeds [23], grape stems, olive seeds [39], Japanese larch treated with concentrated sulfuric acid [40], and coir pith [26].

Regarding the oxidation state of chromium desorbed from chromium-loaded CLB, it was found that the alkaline solutions preferentially desorbed Cr(VI) (Table 1); hence, chromate anions were present on the biosorbent surface. Chromate ions can be easily desorbed by changing the charge of a biosorbent under alkaline conditions. In contrast, the acidic solutions favored the desorption of Cr(III), which is possibly due to the electrostatic repulsion between the Cr(III) cations and the protonated functional groups on the biosorbent surface and/or to the displacement of Cr(III) cations by protons of acidic solutions.

### 2.2. Desorption Kinetics of the Chromium Biosorbed on CLB at Different Concentrations of the Eluent Solutions

**Figure 1:**
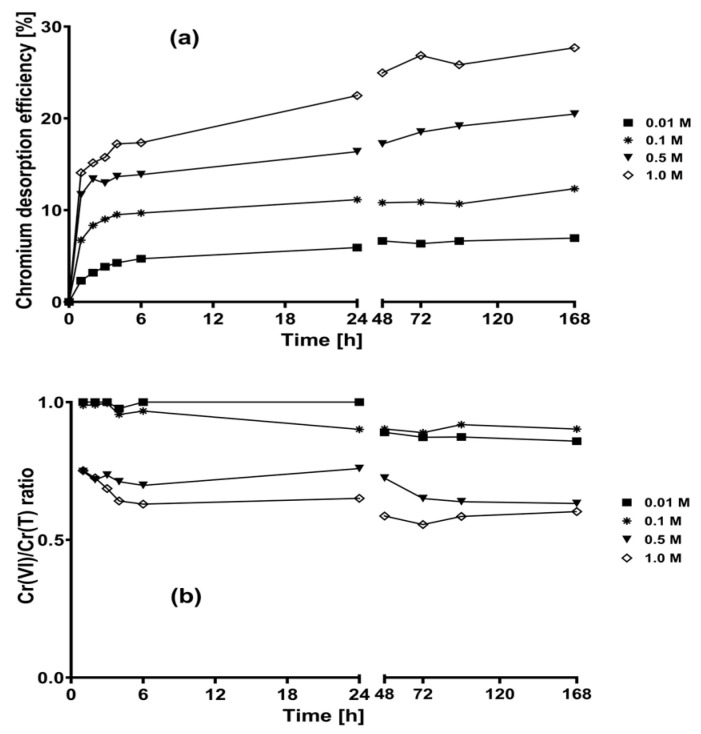
Chromium desorption kinetics of loaded CLB using NaOH eluent solutions. (**a**) Chromium desorption efficiency; (**b**) Cr(VI)/Cr(T) ratio.

Figure 1a shows the total chromium desorption efficiencies obtained when the tested NaOH solutions were put in contact with chromium-loaded CLB. The total chromium desorption efficiencies increased from 6% to 26% as the concentration of the NaOH eluent solutions increased from 0.01 to 1 M. In contrast, the total chromium desorption efficiency of deionized water was negligible (<1%).

The total chromium desorption by the eluting NaOH solutions occurred mainly during the first 6 h of contact. At later time points, chromium desorption efficiency did not significantly increase when 0.01 and 0.1 M NaOH solutions were used. However, chromium desorption continued as contact time increased when 0.5 and 1 M NaOH solutions were applied; this probably happened because the integrity of the biosorbent biomass was lost due to the more alkaline nature of 0.5 M and 1 M NaOH solutions [37]. Therefore, the higher the NaOH concentration, the greater the loss of biomass integrity and the greater the amount of chromium desorbed from chromium-loaded CLB.

Meanwhile, for all the NaOH eluent solutions evaluated, most of the desorbed chromium was Cr(VI), as indicated by the Cr(VI)/Cr(T) ratio > 0.5 (Figure 1b). These results suggest that the desorbed Cr(VI) interacted with the biosorbent through electrostatic attractive forces that were modified by the pH of the eluent solution, favoring the desorption of Cr(VI). However, the amount of chromium anions biosorbed on CLB must have been low; thus, high metal desorption efficiencies were not reached. 

The lowest Cr(VI)/Cr(T) ratios were obtained when 0.5 and 1.0 M NaOH solutions were used, which could be due to biosorbent damage during desorption, causing the release of Cr(III) ions into the solution. Furthermore, the increased amount of Cr(III) ions in the alkaline solutions could possibly be due to the hydrolysis of aqueous Cr(III) at pH close to 12 to yield tetrahydroxochromate(III) (Cr(OH)_4_^−^) anions [41].

The abovementioned results are consistent with the low Cr(VI) desorption percentages that have been obtained in studies of chromium desorption from chromium-loaded tamarind (*Tamarindus indica*) seeds [23] and *Sargassum siliquosum* biomass [24] using eluent NaOH solutions.

In contrast, Cr(VI) recovery percentages higher than 85% have been reported in studies of chromium desorption from chromium-loaded *Chlamydomonas reinhardtii* [42], *Lentinus sajor-caju* [43], *Rhizopus nigricans* immobilized in polysulfone [44], *Mucor hiemalis* [45], amine-modified polyacrylamide-grafted coconut (*Cocos nucifera*) coir pith [46], and *Neurospora crassa* [47] using eluent NaOH solutions. Apparently, in these last studies the high Cr(VI) desorption percentages obtained with the NaOH eluting solutions were due to the fact that the chromium biosorbed on the biosorbents was found only as Cr(VI) or as a mixture of Cr(VI) and Cr(III), wherein Cr(VI) was more dominant. It is worth noting that, except for the amine-modified polyacrylamide-grafted coconut (*Cocos nucifera*) pith, the other biomaterials used in previous studies are not lignocellulose-based biosorbents.

Furthermore, Cr(VI) can be desorbed using alkaline eluent solutions even when the chromium biosorbed on the biosorbents is Cr(III) because the biosorbed Cr(III) can be oxidized to Cr(VI) at high pH values. In this context, Park et al. [29] demonstrated by conducting X-ray photoelectron spectroscopy studies that all the chromium biosorbed on *Aspergillus niger* was present as Cr(III); however, when they used eluent NaOH solutions, some of the chromium eluted was Cr(VI).

**Figure 2:**
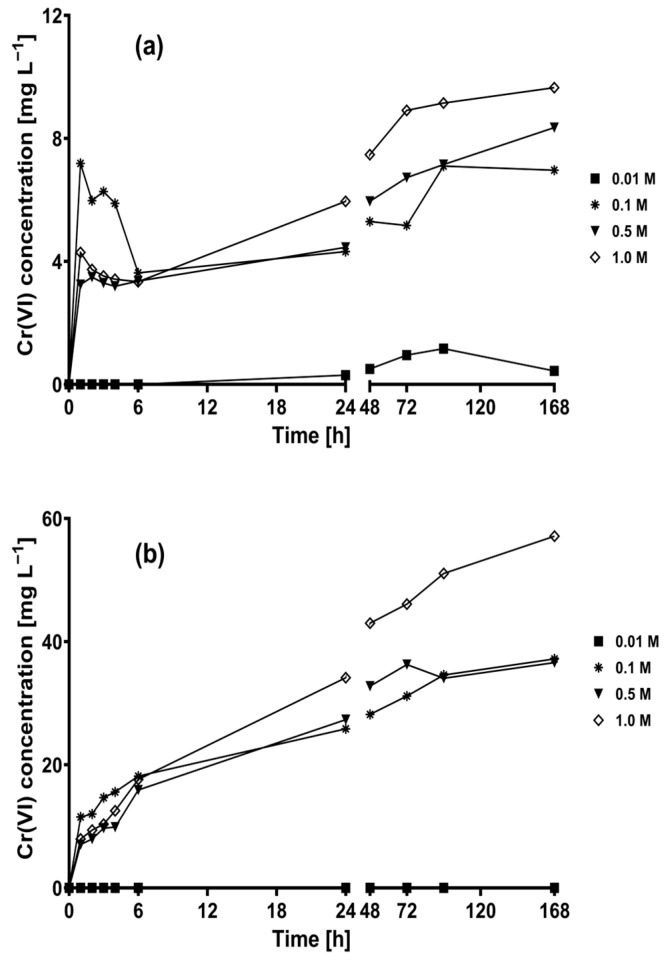
Cr(VI) generation kinetics by CLB under alkaline conditions. (**a**) 100 mg Cr(III) L^−1^; (**b**) 400 mg Cr(III) L^−1^.

In this study, the ability of CLB to oxidize Cr(III) attached to its surface into Cr(VI) under alkaline conditions was determined. Figure 2 shows that the presence of Cr(VI) in the NaOH solution was detected in most of the treatments containing CLB; in contrast, Cr(VI) was not detected in the two CLB-free controls throughout the 168 h duration of the experiments. These results clearly suggest that CLB can oxidize Cr(III) into Cr(VI) under alkaline conditions.

At the first hour of experimentation, Cr(VI) was detected when 0.1, 0.5, and 1 M NaOH solutions containing initial Cr(III) concentrations of 100 mg L^−1^ (Figure 2a) and 400 mg L^−1^ (Figure 2b) were used. The pH of these solutions was higher than 12. When 0.01 M NaOH solution containing 100 mg L^−1^ of Cr(III) was used, Cr(VI) was detected in the solution after 24 h of contact; the slow Cr(VI) generation rate may be due to the pH 8.0 of this NaOH solution. Likewise, Cr(III) was not oxidized into Cr(VI) when 0.01 M NaOH solution with an initial Cr(III) concentration of 400 mg L^−1^ and a pH of 4.0 was used. The above results clearly indicate that CLB is responsible for oxidizing some parts of Cr(III) into Cr(VI) in NaOH solutions with pH ≥ 8.0.

Therefore, NaOH solutions with pH higher than 8.0 provide the desorption conditions favorable for some parts of Cr(III) biosorbed on CLB to be oxidized into Cr(VI). However, it cannot be ruled out that Cr(VI) desorption using NaOH solutions could be the result of the desorption of the chromate oxyanions retained by the biomaterial, as well as the oxidation of part of the Cr(III) biosorbed on CLB into Cr(VI).

**Figure 3:**
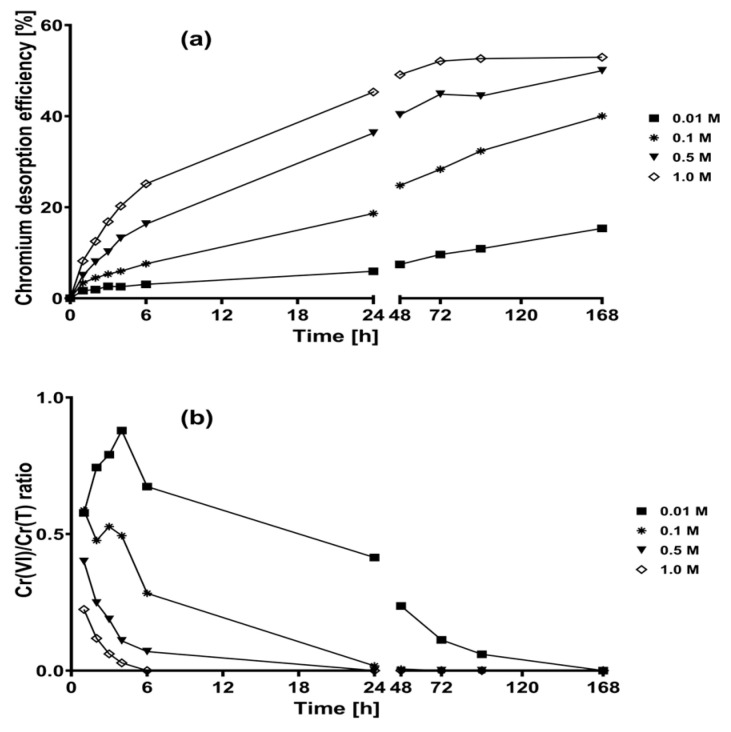
Chromium desorption kinetics of loaded CLB using sulfuric acid eluent solutions. (**a**) Chromium desorption efficiency; (**b**) Cr(VI)/Cr(T) ratio.

Figure 3 shows the percentage of chromium desorption and the Cr(VI)/Cr(T) ratio when different concentrations of H_2_SO_4_ eluent solutions were used. The chromium desorption efficiency gradually increased as the desorption time increased. Likewise, the chromium desorption efficiency at 168 h of experimentation increased from 15.3% to 52.9% as the H_2_SO_4_ concentration increased from 0.01 to 1 M (Figure 3a); notably, these efficiencies were higher than those obtained when NaOH eluent solutions were used.

The most concentrated H_2_SO_4_ solutions (0.5 and 1.0 M) favored the desorption of Cr(III), as evidenced by Cr(VI)/Cr(T) ratios lower than 0.5 throughout the entire experimentation (Figure 3b). In contrast, when 0.01 and 0.1 M H_2_SO_4_ solutions were used, a higher proportion of Cr(VI) was recovered than Cr(III) (Cr(VI)/Cr(T) ratio > 0.5) at contact times less than 6 h; however, this proportion was reversed at later time points. The desorbed chromium was exclusively Cr(III) at 6, 24, 48, and 168 h when 1.0, 0.5, 0.1, and 0.01 M H_2_SO_4_ eluent solutions were used, respectively (Figure 3b). Additionally, none of the evaluated H_2_SO_4_ solutions reduced Cr(VI) into Cr(III) in the absence of CLB.

To explain the above results, the following should be considered: (1) acidic solutions (H_2_SO_4_) protonated the surface of CLB; thus, the electrostatic repulsion between the positively charged biosorbent and the Cr(III) cations biosorbed on CLB could cause the desorption of Cr(III) [34]; (2) the desorption of Cr(VI) by the eluent H_2_SO_4_ solutions could be due to an anion exchange phenomenon between the sulfate ions of H_2_SO_4_ and the chromate ions biosorbed on CLB; and (3) the strongly acidic conditions of the H_2_SO_4_ solutions used during these tests (pH < 1.29) could favor the bioreduction of desorbed Cr(VI) into Cr(III) through the action of the reducing groups present on CLB surface, as reported previously [34].

**Figure 4:**
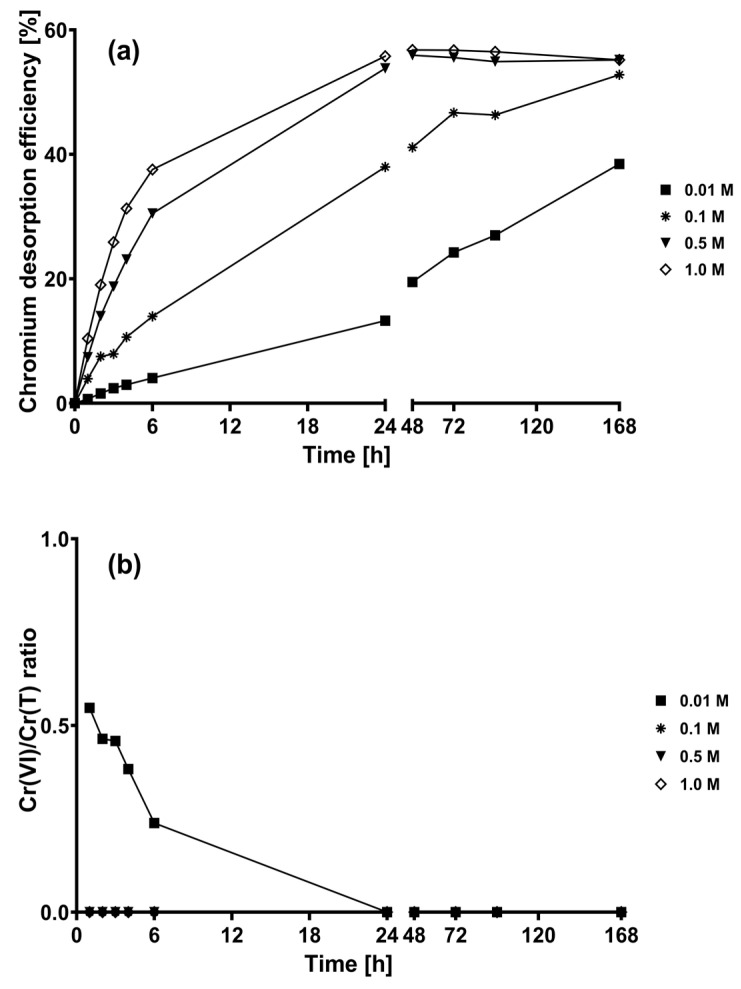
Chromium desorption kinetics of loaded CLB using oxalic acid eluent solutions. (**a**) Chromium desorption efficiency; (**b**) Cr(VI)/Cr(T) ratio.

Figure 4a shows the total chromium desorption percentages when H_2_C_2_O_4_ solutions were used as eluents. When 0.01 and 0.1 M H_2_C_2_O_4_ solutions were used, the chromium desorption efficiency increased throughout the experimentation. At 0.5 and 1 M H_2_C_2_O_4_ solutions, the efficiency increased gradually until 24 h; however, it remained constant at later time points. Likewise, the chromium desorption efficiency at 168 h of contact increased from 38.4% to 55.1% when the H_2_C_2_O_4_ concentration was increased from 0.01 to 1 M. The chromium desorption efficiencies obtained using H_2_C_2_O_4_ were slightly higher than those achieved with similar concentrations of H_2_SO_4_, except for those obtained at 0.01 M.

Moreover, chromium desorption using H_2_C_2_O_4_ solutions was faster than that using H_2_SO_4_ solutions. Since the pH values of H_2_SO_4_ and H_2_C_2_O_4_ eluent solutions were similar, the differences observed in the rate and extent of chromium desorption suggest that another factor aside from pH facilitated chromium desorption by H_2_C_2_O_4_. The higher chromium desorption rates achieved with H_2_C_2_O_4_ solutions may be due to the ability of this dicarboxylic acid to form soluble complexes with Cr(III) [48,49]. Owing to this property, H_2_C_2_O_4_ has been used to extract metals in pieces of wood treated with chromated copper arsenate (CCA) [50,51,52] and chromated copper borate [53]. When chromium, arsenic, and copper were extracted from CCA-treated wood, chromium exhibited the lowest extraction rate; such high resistance to extraction was attributed to the strong bonds established between lignin and chromium [50].

Throughout the total chromium desorption experiments using 0.1, 0.5, and 1 M H_2_C_2_O_4_ solutions, the Cr(VI)/Cr(T) ratios were 0, indicating that all the desorbed chromium was Cr(III) (Figure 4b). However, when 0.01 M H_2_C_2_O_4_ solution was used, a small amount of Cr(VI) was detected during the first 6 h of contact; thereafter, all chromium in the solution was Cr(III).

H_2_C_2_O_4_ has been extensively studied as a Cr(VI)-reducing agent under different environmental conditions [54,55,56]. Likewise, a H_2_C_2_O_4_–Cr(VI) system was used as a prototype in mechanistic studies of oxidation–reduction processes between Cr(VI) and organic substrates [57].

**Figure 5:**
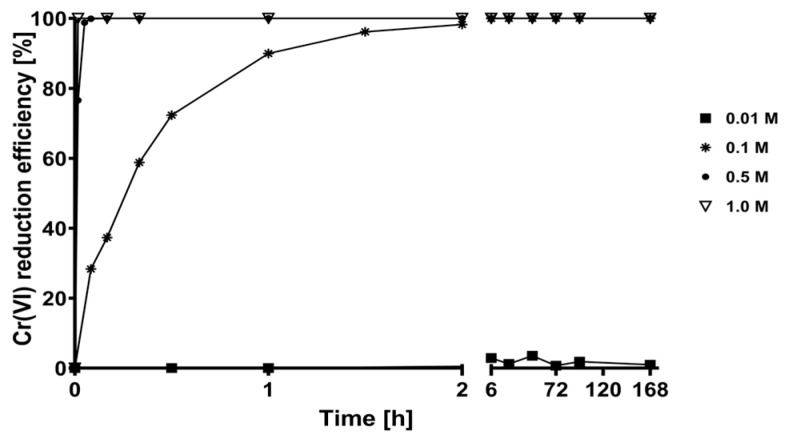
Cr(VI) reduction kinetics by oxalic acid.

Figure 5 shows the kinetic profiles of Cr(VI) reduction efficiency using different concentrations of H_2_C_2_O_4_ solutions in the absence of CLB. The 0.1, 0.5, and 1 M H_2_C_2_O_4_ solutions, which had a pH lower than 1.1, completely reduced 400 mg L^−1^ of Cr(VI) into Cr(III) in less than 3 h of contact. In contrast, 0.01 M H_2_C_2_O_4_ solution at pH 2.36 slowly reduced Cr(VI).

The above results suggest that small amounts of Cr(VI) were biosorbed on CLB; however, the biosorbed Cr(VI) could be bioreduced into Cr(III) more rapidly in the CLB–H_2_C_2_O_4_ desorption systems than in the CLB–H_2_SO_4_ systems due to the high reducing capacity of H_2_C_2_O_4_. Consequently, chromium was recovered mainly as Cr(III) from the first hours of contact.

Therefore, the oxidation state of chromium desorbed from a chromium-loaded biomaterial depends on the chemical nature and concentration of the eluting agent.

### 2.3. Two-Stage Desorption Kinetics of Total Chromium from Loaded CLB

To increase the recovery of chromium, two-stage desorption kinetic experiments were carried out. In these experiments, acidic eluent solutions of H_2_SO_4_ and H_2_C_2_O_4_ were used in the first stage, while alkaline eluent solutions of NaOH were used in the second stage, and vice versa. The contact time between the eluent solutions and the chromium-loaded CLB in each stage was 168 h.

The combinations of acidic eluting solutions (first stage) and alkaline eluting solutions (second stage), as well as alkaline eluting solutions (first stage) and acidic eluting solutions (second stage), are shown in Table 2. This table also displays the chromium desorption efficiencies and the Cr(VI)/Cr(T) ratios achieved in the first and second stages, as well as the total chromium desorption efficiencies achieved in the overall desorption processes. The overall total chromium desorption efficiencies of the two-stage processes were higher than those achieved in the single-stage desorption processes. The highest overall total chromium desorption efficiencies were obtained when the NaOH solutions were used in the first stage and 0.5 M H_2_C_2_O_4_ (79.4–96.1%) and H_2_SO_4_ (59.1–87.4%) solutions were used in the second stage. Likewise, the overall total chromium desorption efficiencies increased as the NaOH concentration in the first stage increased, while the H_2_C_2_O_4_ or H_2_SO_4_ concentrations in the second stage were kept constant at 0.5 M (Table 2).

Meanwhile, the total chromium desorption efficiency only increased between 9.17 and 15.8% when 0.5 M NaOH eluent solution was used in the second stage, resulting in overall chromium desorption efficiencies less than 66.5%. Furthermore, when NaOH eluent solutions were used in the first or second desorption stage, Cr(VI) was detected in the solution (Cr(VI)/Cr(T) > 0); in contrast, only Cr(III) was found in the solution (Cr(VI)/Cr(T) = 0) when acidic eluting solutions (H_2_SO_4_ or H_2_C_2_O_4_) were used in the first or second desorption stage (Table 2).

**Figure 6:**
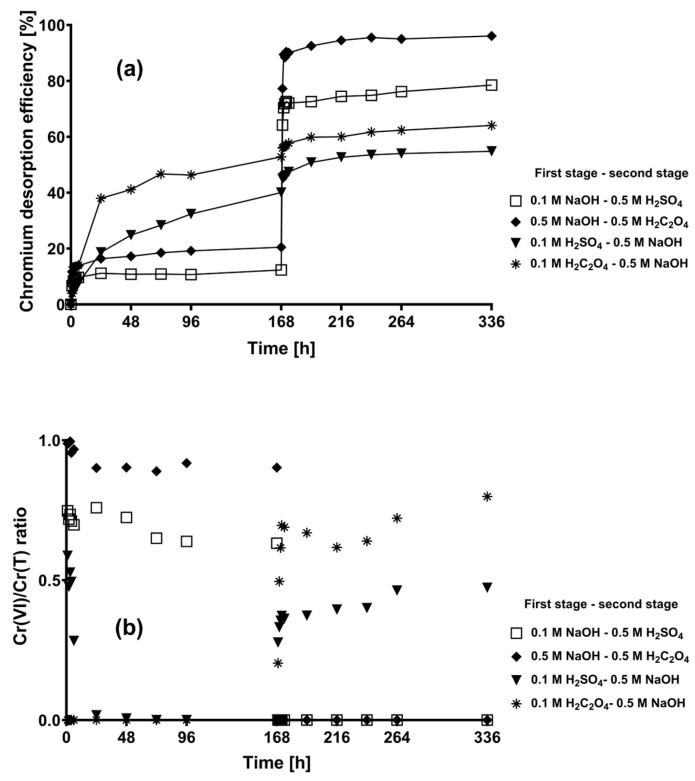
Two-stage chromium desorption kinetics of chromium-loaded CLB throughout the desorption duration of 168 h in each stage. (**a**) Chromium desorption efficiency; (**b**) Cr(VI)/Cr(T) ratio.

Figure 6 shows the kinetic profiles of the overall total chromium desorption efficiencies and Cr(VI)/Cr(T) ratios of several total chromium desorption processes conducted in two stages. It is apparent that the total chromium desorption efficiencies achieved in the first stage using NaOH eluent solutions were the lowest, and the efficiencies subsequently increased when H_2_C_2_O_4_ or H_2_SO_4_ eluent solutions were used in the second stage. In contrast, the chromium desorption efficiencies were higher when acidic eluting solutions were used than when alkaline eluting solutions were used in the first stage; however, the efficiencies did not significantly increase when NaOH eluent solutions were used in the second stage. The highest overall total chromium desorption efficiencies were achieved when 0.5 M NaOH was used in the first stage and 0.5 M H_2_SO_4_ or 0.5 M H_2_C_2_O_4_ was used in the second stage.

In general, total chromium desorption in the second stage occurred mainly during the first 3 h of contact between chromium-loaded CLB and the eluent solution. Furthermore, after a long desorption period, the structural integrity of CLB weakened and it suffered damage when 0.5 M NaOH solution was used in the second stage. This result suggests that chromium desorption should be conducted within a short contact period.

Regarding the oxidation state of the desorbed chromium from chromium-loaded CLB, Cr(VI) was mainly recovered when NaOH eluting solutions were used, as evidenced by the Cr(VI)/Cr(T) ratio > 0.5 (Figure 6b). Likewise, when NaOH was used as the primary eluent (in the first stage), the Cr(VI)/Cr(T) ratios remained almost constant throughout the first elution stage (Figure 6b). Furthermore, when NaOH was used as the secondary eluent (in the second stage), the Cr(VI)/Cr(T) ratios remarkably increased during the first hours of contact. These results may be due to the fact that the pH of the NaOH eluting solutions was higher than 8.0, thus favoring the oxidation of Cr(III) into Cr(VI). In contrast, when acidic solutions were used as the secondary eluent (in the second stage), only Cr(III) was recovered (Cr(VI)/Cr(T) = 0.0). 

**Figure 7:**
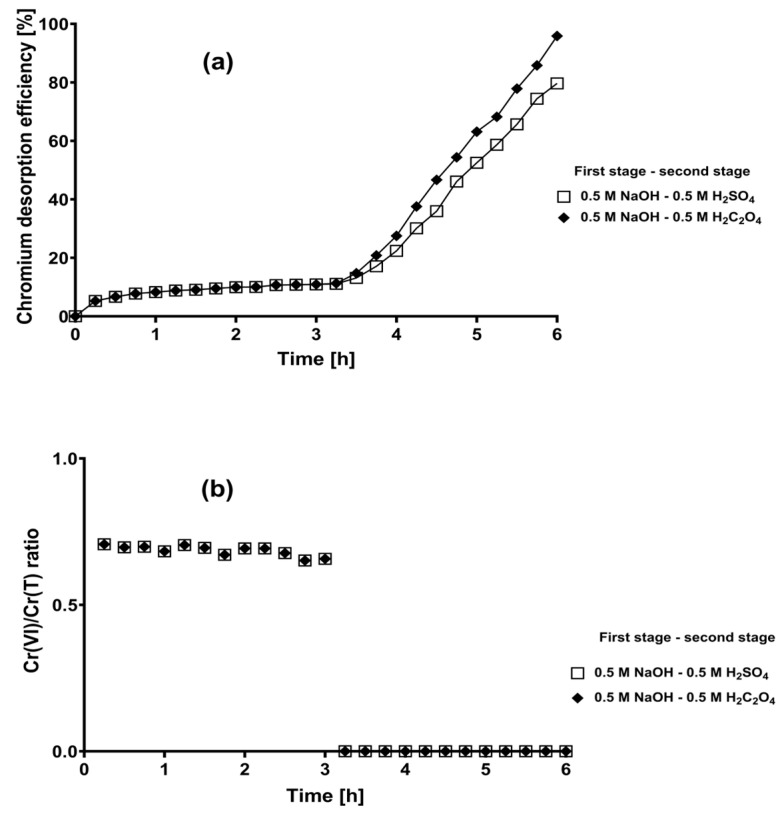
Two-stage chromium desorption kinetics of chromium-loaded CLB throughout a desorption duration of 3 h in each stage. (**a**) Chromium desorption efficiency; (**b**) Cr(VI)/Cr(T) ratio.

Based on these results, chromium desorption kinetics were performed with contact times of 3 h for each elution stage. The combinations of eluting solutions tested were 0.5 M NaOH–0.5 M H_2_C_2_O_4_ and 0.5 M NaOH–0.5 M H_2_SO_4_ because they exhibited the highest overall total chromium desorption efficiencies in the previous experiments. The kinetic profiles of metal desorption are shown in Figure 7.

Chromium desorption by 0.5 M NaOH solution in the first stage started immediately and occurred mainly during the first 30 min of contact; in contrast, chromium desorption by acidic eluents in the second stage maintained an almost constant rate from the first 15 min of contact until the end of desorption (Figure 7).

The chromium desorption efficiency in the first stage with 0.5 M NaOH was low (10.9%); however, it was only slightly lower than that obtained with the same eluent solution at 168 h of contact time. Subsequently, the efficiency gradually increased up to 79.6 and 95.9% in the second stage with 0.5 M H_2_SO_4_ and 0.5 M H_2_C_2_O_4_ as eluents, respectively. It should be noted that the reduction in the contact time from 168 to 3 h in each desorption stage did not have a significant effect on the overall total chromium desorption efficiency of the 0.5 M NaOH–0.5 M H_2_C_2_O_4_ system; however, a slight decrease in the metal desorption efficiency, with respect to that obtained at the total contact time of 336 h, was observed when the eluent solutions 0.5 M NaOH and 0.5 M H_2_SO_4_ were used in the first and second stages, respectively (Table 2 and Figure 7).

Regarding the oxidation state of the desorbed chromium from loaded CLB, the NaOH solution in the first elution stage favored the recovery of mixtures of Cr(VI) and Cr(III), where Cr(VI) dominated (Cr(VI)/Cr(T) > 0.5), while the acidic eluent solutions in the second elution stage favored the desorption of chromium as Cr(III) (Cr(VI)/Cr(T) ratio = 0) (Table 2 and Figure 7). On the other hand, when elution was conducted with total contact times of 6 h, no damage in biological material was detected, contrary with that observed in some of the desorption trials conducted with total contact times of 336 h.

From the above, the main novelties of our work can be summarized as follows: (1) For the first time in the literature, a two-stage desorption process for the efficient recovery of chromium was described. (2) It was demonstrated that the order of the eluent solutions in a two-stage process significantly affects chromium recovery, and (3) it was demonstrated that the oxidation state of the recovered chromium depends on the chemical nature and concentration of the eluent solution.

## 3. Materials and Methods

### 3.1. Chemical Reagents

A 100 mM Cr(VI) stock solution was prepared with potassium chromate (K_2_CrO_4_). The Cr(VI) stock solution was diluted with deionized water to prepare 15 mM (800 mg L^−1^) Cr(VI) test solution. The following are the eluting solutions used in this study: sodium sulfate (Na_2_SO_4_), potassium sulfate (K_2_SO_4_), sodium nitrate (NaNO_3_), potassium chloride (KCl), sodium chloride (NaCl), calcium chloride (CaCl_2_), sodium oxalate (Na_2_C_2_O_4_), sodium citrate (Na_3_C_6_H_5_O_7_•2H_2_O), tartaric acid (C_4_H_6_O_6_), oxalic acid (H_2_C_2_O_4_•2H_2_O), citric acid (C_6_H_8_O_7_•H_2_O), boric acid (H_3_BO_3_), hydrochloric acid (HCl), sulfuric acid (H_2_SO_4_), nitric acid (HNO_3_), acetic acid (C_2_H_4_O_2_), potassium hydroxide (KOH), sodium hydroxide (NaOH), potassium carbonate (K_2_CO_3_), sodium carbonate (Na_2_CO_3_), sodium bicarbonate (NaHCO_3_), ethylenediaminetetraacetic acid (EDTA), phosphate buffer of pH 7.0, and phthalate buffer of pH 4.0. Analytical-grade chemical reagents were used in this study (J.T. Baker, Estado de México, Mexico). Deionized water was used as the control eluent solution.

### 3.2. Biomaterial

CLB particles with a size of 0.42–0.5 mm were obtained according to the procedure outlined by Netzahuatl-Muñoz et al. [34]. To saturate the biosorbent with chromium, 1 g L^−1^ CLB particles was mixed with the Cr(VI) test solution at pH 1.5 for 72 h. Afterward, Cr(VI)-loaded CLB particles were rinsed with deionized water, dried at 60 °C for 48 h, and subsequently analyzed to determine the amount of chromium they contained.

### 3.3. Chromium Desorption Studies

#### 3.3.1. Selection of Eluting Solutions

To determine the influence of the eluting solutions on chromium desorption from chromium-loaded CLB, 2 [g L^−1^] dry weight of chromium-loaded CLB was placed in glass flasks containing 20 mL of the assayed eluting solutions. The flasks were kept under constant agitation at 150 rpm for 3 h at room temperature (approximately 28 ± 2 °C). Subsequently, the CLB particles were separated from the eluting solutions by centrifugation at 3500 rpm for 5 min, and the supernatants were analyzed to determine their Cr(VI) and total chromium concentrations. The eluting solutions that exhibited the highest chromium desorption efficiencies were selected for further studies.

#### 3.3.2. Chromium Desorption Kinetic Studies in Single and Two Stages

After choosing the best eluting solutions for chromium desorption, single-stage desorption kinetic experiments were performed to determine their optimal concentrations. In 500 mL Erlenmeyer flasks, chromium-loaded CLB samples (2 g L^−1^) were put in contact with 100 mL of the previously selected eluting solutions at 0.01, 0.1, 0.5, and 1.0 M concentrations. The flasks were agitated continuously at 150 rpm and maintained at room temperature (28 ± 2 °C). Samples were collected after 1, 2, 3, 4, 6, 24, 48, 72, 96, and 168 h of contact between the chromium-loaded CLB and eluting solution.

For the two-stage desorption kinetic experiments, the CLB particles that were previously in contact with a preselected eluting solution for 168 h during the first elution stage were separated from the eluting solution via centrifugation at 3500 rpm for 5 min, washed with deionized water, and subsequently placed in 500 mL Erlenmeyer flasks containing 100 mL of the second eluting solution at a given concentration. The flasks were continuously shaken at 150 rpm at 28 ± 2 °C. The samples were collected after 1, 2, 3, 4, 6, 24, 58, 92, 116, and 168 h of desorption during the second stage, which corresponded to total desorption durations (from the beginning of the first desorption stage) of 169, 170, 171, 172, 174, 192, 226, 260, 284, and 336 h. Additionally, a procedure similar to the one described above was followed to obtain more detailed data on the chromium desorption kinetics in two stages; however, the desorption time in each stage was 3 h, with sampling every 15 min.

The samples collected in the single- and two-stage chromium desorption kinetic experiments were centrifuged at 3500 rpm for 5 min, and the Cr(VI) and total chromium concentrations in the supernatants obtained were quantified.

#### 3.3.3. Cr(III) Oxidation Assays by CLB in NaOH Solutions

To determine the possible oxidation of Cr(III) to Cr(VI) by CLB under alkaline conditions, kinetic experiments were performed in which CLB samples were mixed with 0.01, 0.1, 0.5, or 1.0 M NaOH solutions containing Cr(III) ions at 100 or 400 mg L^−1^. The resulting suspensions were kept under the same operating conditions used in the desorption kinetic studies. Samples were withdrawn at different experimental times and then centrifuged at 3500 rpm for 5 min; the supernatants obtained were used to quantify the Cr(VI) concentration of the samples. CLB-free controls containing only Cr(III) ions in NaOH solutions and CLB-free controls containing only deionized water and Cr(III) ions were simultaneously tested under the aforementioned experimental conditions.

#### 3.3.4. Cr(VI) Reduction Assays by Oxalic Acid (H_2_C_2_O_4_) at Different Concentrations

To determine if H_2_C_2_O_4_ could reduce Cr(VI) to Cr(III) in the absence of CLB, 0.01, 0.1, 0.5, and 1.0 M H_2_C_2_O_4_ solutions containing 400 mg L^−1^ of Cr(VI) ions were prepared. The resulting suspensions were kept under the same operating conditions used in the desorption kinetic studies. Samples were withdrawn at different experimental times, and their Cr(VI) concentrations were immediately analyzed.

### 3.4. Analytical Methods

#### 3.4.1. Determination of Cr(VI), Cr(III), and Total Chromium Concentrations in Solution

Photocolorimetric methods facilitated using a Thermo Scientific Evolution^™^ 201 spectrophotometer (Thermo Fisher Scientific, Waltham, MA, USA) were conducted to determine the Cr(VI) and total chromium concentrations in solutions. Cr(VI) concentration was quantified using the 1,5-diphenylcarbohydrazide method, while the total chromium concentration was measured via performing the alkaline hypobromite oxidation method according to the procedures outlined in Hach methods 8023 and 8024, respectively, of the Hach Water Analysis Handbook [58].

Standard calibration curves were used to quantify the Cr(VI) and total chromium concentrations in solutions. Cr(III) concentrations in solutions were estimated using the difference between the Cr(VI) and total chromium concentrations [1,58].

#### 3.4.2. Determination of Total Chromium Concentration in Chromium-Loaded Biosorbent

To quantify the total chromium concentration in the chromium-loaded CLB samples, a certain amount of the chromium-loaded biosorbent was digested in a Digesdahl^®^ digester [58]. During the digestion, the solid samples were oxidized with a mixture of concentrated H_2_SO_4_ and 30% H_2_O_2_. H_2_SO_4_ dehydrated and carbonized the sample, and the addition of H_2_O_2_ completed its decomposition through the action of peroxymonosulfuric acid (H_2_SO_5_), which was produced at 440 °C—the temperature at which the digestion procedure was carried out [58]. Alkaline hypobromite oxidation was performed to determine the total chromium concentration of the resulting solution.

#### 3.4.3. Estimation of Chromium Desorption Efficiency

The total chromium desorption efficiency (% *RCr*) was estimated using the following mass balance formula [59]:

(1) $$\;\%\;RCr=\frac{Cr{\left(T\right)}_{s}V}{Cr{\left(T\right)}_{0}M}\times 100,$$

where *Cr*(*T*)*_s_* is the concentration of total chromium in the eluting solution (mg L^−1^), *V* is the volume of the eluting solution (L), *Cr*(*T*)_0_ is the initial concentration of chromium in the chromium-loaded CLB (mg g^−1^), and *M* is the mass of CLB saturated with heavy metal (g). The calculation of chromium desorption efficiencies in the kinetic experiments was corrected considering the heavy metal concentration in the eluent solution and the volume of solution withdrawn from each sample.

#### 3.4.4. Predominant Oxidation State of Recovered Chromium in the Eluting Solution

To ascertain the predominant oxidation state of chromium desorbed by the eluting solutions after the contact between chromium-loaded CLB and the eluting solutions, the fraction of *Cr(VI)* in the eluting solution (*Cr*(*VI*)*/Cr*(*T*)) was determined using the following ratio:

(2) $$Cr\left(VI\right)/Cr\left(T\right)=\frac{Cr{\left(VI\right)}_{s}}{Cr{\left(T\right)}_{s}}$$

where *Cr*(*VI*)_s_ and *Cr*(*T*)*_s_* are the concentrations of *Cr*(*VI*) and total chromium in the eluting solution, respectively. *Cr*(*VI*)*/Cr*(*T*) ratios higher than 0.5 indicate a greater proportion of *Cr*(*VI*) in the eluting solution, while ratios less than 0.5 indicate a greater proportion of Cr(III) in the eluting solution.

## 4. Conclusions

The chromium biosorbed on CLB exhibited great resistance to desorption by acidic and alkaline eluent solutions, which is a behavior that has been previously reported with other biomaterials. Chromium biosorbed on CLB was mainly desorbed as Cr(III) using acidic eluting solutions (pH < 1.1) and as Cr(VI) with alkaline eluting solutions (pH > 8). The desorption of Cr(VI) by alkaline solutions was due to the presence of Cr(VI) biosorbed on CLB and the ability of CLB to oxidize part of the Cr(III) biosorbed on its surface into Cr(VI) under alkaline conditions. Likewise, CLB was capable of bioreducing Cr(VI) into Cr(III) at very low pH values. Consequently, the oxidation state of the recovered chromium depended on the chemical nature and concentration of the eluent solution. This study demonstrated for the first time that using 0.5 M NaOH eluent solution in the first desorption stage and 0.5 M H_2_C_2_O_4_ eluent solution in the second desorption stage facilitated the recovery of almost all chromium initially bound to CLB (overall desorption efficiency = 95.9%), even within a short desorption period (6 h). Through this process, chromium was mainly recovered as Cr(III). The results also suggest the possible regeneration of chromium-loaded CLB for its subsequent use in other biosorption/desorption cycles.

## Figures and Tables

**Table 1:** Effect of eluent solutions on the total chromium desorption efficiency and Cr(VI)/Cr(T) ratio.

Eluent Solution	Chromium Desorption Efficiency (%)	Cr(VI)/Cr(T) Ratio
Deionized water	0.96	0.927
EDTA (0.1 M)	1.57	0.932
Sodium sulfate (0.1 M)	1.07	0.966
Potassium sulfate (0.1 M)	1.67	0.965
Sodium nitrate (0.1 M)	1.65	0.904
Potassium chloride (0.1 M)	1.38	0.917
Sodium chloride (0.1 M)	1.30	0.907
Calcium chloride (0.1 M)	1.69	0.891
Sodium oxalate (0.1 M)	1.90	0.929
Sodium citrate (0.1 M)	1.59	0.835
Phosphate buffer (pH 7.0)	2.08	0.942
Phtalate buffer (pH 4.0)	1.92	0.925
Tartaric acid (0.1 M)	2.31	0.000
Oxalic acid (0.1 M)	10.10	0.000
Citric acid (0.1 M)	1.66	0.494
Boric acid (0.1 M)	1.19	0.870
Hydrochloric acid (0.1 M)	2.75	0.772
Sulfuric acid (0.1 M)	7.04	0.623
Nitric acid (0.1 M)	3.02	0.713
Acetic acid (0.1 M)	1.00	0.886
Potassium hydroxide (0.01 M)	5.32	0.962
Potassium hydroxide (0.1 M)	12.60	0.932
Sodium hydroxide (0.01 M)	5.32	0.905
Sodium hydroxide (0.1 M)	13.00	0.863
Potassium carbonate (0.01 M)	3.66	0.919
Potassium carbonate (0.1 M)	6.63	0.860
Sodium carbonate (0.01 M)	4.15	0.944
Sodium carbonate (0.1 M)	6.36	0.930
Sodium bicarbonate (0.01 M) Sodium bicarbonate (0.1 M)	2.18 3.05	0.925 0.938

**Table 2:** Chromium desorption efficiency achieved in single and two stages.

First Desorption Stage (Contact Time: 168 h)			Second Desorption Stage (Contact Time: 168 h)			Overall Desorption Process
Eluent Solution	Chromium Desorption Efficiency (%)	Cr(VI)/Cr(T) Ratio	Eluent Solution	Chromium Desorption Efficiency (%)	Cr(VI)/Cr(T) Ratio	Chromium Desorption Efficiency (%)
Oxalic acid (0.01 M)	38.4	0.00	Sodium hydroxide (0.5 M)	15.8	0.540	54.2
Oxalic acid (0.1 M)	52.8	0.00	Sodium hydroxide (0.5 M)	11.3	0.811	64.1
Oxalic acid (0.5 M)	55.2	0.00	Sodium hydroxide (0.5 M)	11.1	0.799	66.3
Oxalic acid (1.0 M)	55.2	0.00	Sodium hydroxide (0.5 M)	9.17	0.699	64.4
Sulfuric acid (0.01 M)	15.3	0.00	Sodium hydroxide (0.5 M)	14.6	0.435	29.9
Sulfuric acid (0.1 M)	40.0	0.00	Sodium hydroxide (0.5 M)	14.9	0.473	54.9
Sulfuric acid (0.5 M)	50.0	0.00	Sodium hydroxide (0.5 M)	11.3	0.683	61.3
Sulfuric acid (1 M)	52.9	0.00	Sodium hydroxide (0.5 M)	11.1	0.605	64.0
Sodium hydroxide (0.01 M)	6.96	0.858	Oxalic acid (0.5 M)	72.4	0	79.4
Sodium hydroxide (0.1 M)	12.3	0.902	Oxalic acid (0.5 M)	81.2	0	93.5
Sodium hydroxide (0.5 M)	20.5	0.632	Oxalic acid (0.5 M)	75.6	0	96.1
Sodium hydroxide (0.01 M)	6.96	0.858	Sulfuric acid (0.5 M)	52.1	0	59.1
Sodium hydroxide (0.1 M)	12.3	0.902	Sulfuric acid (0.5 M)	66.2	0	78.5
Sodium hydroxide (0.5 M)	20.5	0.632	Sulfuric acid (0.5 M)	66.9	0	87.4
First desorption stage (contact time: 3 h)			Second desorption stage (contact time: 3 h)			Overall desorption process
Eluent Solution	Chromium Desorption Efficiency (%)	Cr(VI)/Cr(T) Ratio	Eluent Solution	Chromium Desorption Efficiency (%)	Cr(VI)/Cr(T) Ratio	Chromium Desorption Efficiency (%)
Sodium hydroxide (0.5 M)	10.9	0.657	Oxalic acid (0.5 M)	85.0	0	95.9
Sodium hydroxide (0.5 M)	10.9	0.657	Sulfuric acid (0.5 M)	68.7	0	79.6

## Data Availability

All relevant data are within the paper.
